# Liver Fibrosis and Mechanisms of the Protective Action of Medicinal Plants Targeting Inflammation and the Immune Response

**DOI:** 10.1155/2015/943497

**Published:** 2015-04-14

**Authors:** Florent Duval, Jorge E. Moreno-Cuevas, María Teresa González-Garza, Carmen Maldonado-Bernal, Delia Elva Cruz-Vega

**Affiliations:** ^1^Catedra de Terapia Celular, Escuela de Medicina, Tecnológico de Monterrey, Avenida Morones Prieto 3000 Pte., 64710 Monterrey, NL, Mexico; ^2^Laboratorio de Investigación en Inmunología y Proteómica, Hospital Infantil de México Federico Gómez, Calle Dr. Márquez 162, 06720 Ciudad de México, DF, Mexico

## Abstract

Inflammation is a central feature of liver fibrosis as suggested by its role in the activation of hepatic stellate cells leading to extracellular matrix deposition. During liver injury, inflammatory cells are recruited in the injurious site through chemokines attraction. Thus, inflammation could be a target to reduce liver fibrosis. The pandemic trend of obesity, combined with the high incidence of alcohol intake and viral hepatitis infections, highlights the urgent need to find accessible antifibrotic therapies. Medicinal plants are achieving popularity as antifibrotic agents, supported by their safety, cost-effectiveness, and versatility. The aim of this review is to describe the role of inflammation and the immune response in the pathogenesis of liver fibrosis and detail the mechanisms of inhibition of both events by medicinal plants in order to reduce liver fibrosis.

## 1. Introduction

Fibrosis is an inappropriate tissue repair of the liver resulting from almost all of the chronic liver injuries including alcohol induced damage, chronic viral hepatitis (HBV and HCV), autoimmune, parasitic, and metabolic diseases, and less frequently toxic or drugs exposure [1]. When fibrosis is not controlled, it can further progress into cirrhosis. In contrast with the traditional idea that cirrhosis is an irreversible state, there is solid evidence indicating that fibrosis even cirrhosis could be reversible [2].

Liver fibrosis is an important public health concern with significant morbidity and mortality [3]. Hundreds of millions of people worldwide suffer from cirrhosis [4]. Chronic viral hepatitis B and C, alcoholic liver diseases, and nonalcoholic fatty liver diseases are the three most common causes [5]. Prevalence of chronic liver diseases, hence hepatic fibrosis-cirrhosis, is predicted to increase, due in part to the rising prevalence of obesity and metabolic syndrome, especially in developed countries [6].

Pathogenesis of liver fibrosis is complex and varies between different kinds of hepatic injuries. Usually after acute liver damage, parenchymal cells regenerate and replace the necrotic and apoptotic cells; this process is associated with an inflammatory response and a limited deposition of extracellular matrix. When injury persists, eventually the regenerative response fails and hepatocytes are substituted by abundant extracellular matrix mainly composed by collagen type I-III-IV, fibronectin, elastin, laminin, and proteoglycans. Activated hepatic stellate cells (HSCs) are the main sources of extracellular matrix [7].

Inflammation is an important and complex feature of liver fibrosis. Following liver injury, an accumulation of recruited inflammatory cells in the injurious site occurs. Cells from innate immune response, including, platelets, neutrophils, macrophages, mast cells, and natural killer (NK) cells, and from the adaptive immune response, such as T- and B-cells, participate in the fibrogenesis process. A wide repertoire of pro- and anti-inflammatory compounds, which encompasses cytokines, chemokines, growth factors, and products of oxidative stress, mediates the inflammatory response of immune cells during the fibrosis process [8]. HSCs also take part actively in the inflammation process through interaction with diverse types of immune cells [9]. Furthermore, HSCs conversion from a quiescent to an activated state characterized by a myofibroblast-like phenotype responsible for proliferation and excessive extracellular matrix deposition is regulated by inflammatory mediators, including transforming growth factor-beta (TGF-*β*) and tumor necrosis factor-alpha (TNF-*α*) [7].

There is no standard treatment for liver fibrosis, although it is known that reducing liver injury events, such as interruption of alcohol intake or successful treatment of viral hepatitis, contributes to control of the process. Nevertheless, these actions do not seem to be sufficient in the vast majority of patients to avoid progression to cirrhosis [8]. Even though important advances have been made in the knowledge of the pathogenesis of hepatic fibrosis for the past 20 years, there are still important gaps to translate this basic information into efficient antifibrotic drugs. Treatment strategies for liver fibrosis should take into account the versatility of its pathogenesis and acting on all the events involved starting with inflammation.

Supported by their safety, cost-effectiveness, and versatility, medicinal plants enjoy a growing popularity as antifibrotic agents. We already reviewed how medicinal plants reduce liver fibrosis by inhibiting HSCs activation and reducing ECM deposition [10]. However, other antifibrotic mechanisms could explain this activity such as suppression of inflammation and the immune response. This review focuses on another way bioactive compounds from thirteen known hepatoprotective plants, including* Curcuma longa*,* Silybum marianum*,* Ginkgo biloba*,* Salvia miltiorrhiza*,* Glycyrrhiza glabra*,* Scutellaria baicalensis*,* Bupleurum falcatum*,* Phyllanthus* species,* Berberis aristata*,* Picrorrhiza kurroa*,* Ginseng* species,* Andrographis paniculata*, and coffee species, reduce liver fibrosis: the suppression of inflammation and the immune response.

## 2. Role of Inflammation and Immune Response in the Pathogenesis of Liver Fibrosis

### 2.1. Platelets

Platelets are among the first cells recruited to the injurious site. Platelets initiate coagulation cascade to limit blood loss converting fibrinogen into fibrin [8]. Involvement of platelets in the fibrogenesis results from its capacity to release cytokines like TGF-*β* and platelet derived growth factor (PDGF) [11]. Platelets also produce serotonin, which mediates liver regeneration [12]. Additionally, platelets release the platelet derived chemokine (C-X-C) ligand 4 (CXCL4), also known as platelet factor 4 (PF4) [13]. Patients with advanced hepatitis C virus-induced fibrosis or nonalcoholic steatohepatitis, as well as animal models of liver fibrosis induced by carbon tetrachloride (CCl_4_) and thioacetamide (TAA), have increased intrahepatic levels of CXCL4 suggesting a role of CXCL4 in the fibrogenic process. This role were further exposed by observing that CXCL4(−/−) mice have reduced liver damage and changes in the expression of fibrosis-related genes, including matrix metalloproteinase (MMP)-9, tissue inhibitor of metalloproteinases (TIMP)-1, TGF-*β*1, and interleukin (IL)-10, as well as a decreased infiltration of neutrophils and CD8+ T cells into the liver. In the same study, CXCL4 stimulated the proliferation, chemotaxis, and chemokine expression of HSCs* in vitro* [14].

### 2.2. Neutrophils

During liver injury, neutrophils rapidly infiltrate and transmigrate into the hepatic parenchyma. Transmigration is a chemokine-mediated event that involves adhesion molecules such as integrin and intracellular adhesion molecule-1 (ICAM-1). Next, neutrophils adhere to hepatocytes through hepatocyte ICAM-1 and *β*2 integrins and neutrophil Mac-1 (CD11b/CD18). Contact between hepatocytes and neutrophils triggers formation of reactive oxygen by nicotinamide adenine dinucleotide phosphate (NADPH) oxidase and release of proinflammatory proteases through degranulation leading to killing of hepatocytes, one of the fibrogenic stimuli [15]. Besides, neutrophils synthesize human neutrophil peptide-1 (HNP-1) and IL-17A, which enhance hepatic fibrosis by inducing cell proliferation [16] and activating HSCs [17], respectively. Implication of neutrophils in inflammation also involves reactive oxygen formation [18]. However, importance of neutrophils in the fibrogenic process has not always been clear. Indeed, bile duct-ligated rats depleted of neutrophils showed no difference in hepatic fibrogenesis compared with control rats [19]. Neutrophils have been also associated with liver repair since neutrophils depletion blocks early collagen degradation in repairing cholestatic rat livers [20].

### 2.3. Mast Cells

Mast cells are immune cells involved in immunoglobulin E- (IgE-) associated immediate hypersensitivity and allergic disorders and in various liver diseases. They are naturally present in the liver [21, 22]. These cells could play a role in the development of liver fibrosis as suggested by the correlation between the increased number of mast cells and the amount of liver fibrosis in chronic liver diseases such as primary biliary cirrhosis and alcoholic liver diseases [22]. Nevertheless, this idea is controversial. Studies based on mast cell-deficient mutant Ws/Ws rats and mice did not find any important role for mast cells in the development of liver fibrosis [23, 24]. However, mast cells elaborate a wide range of mediators that have been associated with different activities in liver fibrosis, including tryptase, chymase proteases, interleukins (IL-3, IL-4, IL-5, IL-6, IL-9, IL-10, IL-11, IL-12, IL-13, IL-15, IL-16, IL-18, IL-25, and TNF-*α*), chemokines (macrophage inflammatory protein-1*α* (MIP-1*α*)), hematopoietic factors (granulocyte macrophage colony stimulating factor (GM-CSF)), stem cell factor (SCF), TGF-*β*, vascular endothelial growth factor (VEGF), nerve growth factor (NGF), several MMPs, heparin, histamine, chondroitin sulfates, cathepsin, carboxypeptidases, and peroxidase [21]. The chymase has been linked with the production of angiotensin II and the development of myocardium and renal fibrosis [21] while mast cell tryptase induces proliferation, migration, and synthesis of collagen type I by fibroblasts [25, 26].

### 2.4. Natural Killer

NK cells are involved in defending host against pathogens like hepatitis C virus by recruiting virus-specific T cells and inducing antiviral immunity in liver [9]. Liver has a rich population of NK cells. Upon liver injury, NK cells accumulate through chemokine receptor CXCR6 dependent pathway exacerbating the inflammatory response and promoting hepatic fibrogenesis [27]. The influence of NK cells, especially the CD1d-restricted natural killer T (NKT) cells, on the fibrogenic response has been observed by using mice lacking mature NKT cells caused by genetic disruption of the CD1d molecule. CD1d-knockout mice developed minimal hepatic fibrosis induced by administration of TAA, which was accompanied by reduction in collagen type I alpha 1 (COL1A1) and TIMP-1 expression [28]. Other molecules have been identified as contributors of the profibrogenic effect of NK cells. Mice fed a methionine-choline-deficient (MCD) diet, an animal model of nonalcoholic fatty liver disease, and depleted in NKT cells have significantly attenuated hedgehog and osteopontin expression and fibrosis suggesting that NKT cells promote fibrogenesis via osteopontin and hedgehog pathways [29]. Profibrogenic activity of NK cells also comes from its ability to produce proinflammatory cytokines such as IL-4 and IL-13 [30]. Additionally, NK cells kill hepatocytes through release of tumor necrosis factor-related apoptosis-inducing ligand (TRAIL) and/or granzyme B leading to liver injury that could further progress in liver fibrosis. Nevertheless, NK and NKT cells have also drawn attention to inhibiting liver fibrosis [31]. NK cells and NKT reduce liver fibrosis by producing interferon gamma (IFN-*γ*) and inducing death of early or senescence of activated HSCs [32, 33]. NK cells kill activated HSCs via retinoic acid early inducible 1/NKG2D dependent, TRAIL dependent, and Fas ligand dependent mechanisms, thereby ameliorating liver fibrosis [34, 35]. The pattern of increased levels of NK cell-activating ligands (ribonucleic acid export 1 (RAE-1) in mice, MHC class I polypeptide-related sequence A (MICA) in human) and TRAIL receptors is not observed in quiescent and fully activated HSCs, making them resistant to such killing [31, 32]. Antifibrotic effect of NK cells can also be explained by the production of IFN-*γ*, which is a cytokine that directly inhibits HSCs activation leading to reduced liver fibrosis [36]. Additionally, IFN-*γ* induces HSCs apoptosis in a signal transducer and activator of transcription 1 (STAT1) dependent manner, which inhibits HSCs proliferation, attenuates TGF-*β* signaling, and stimulates NK cell cytotoxicity towards HSCs, or upregulating NKG2D and TRAIL expression on NK cells [34, 37].

### 2.5. Macrophages

Macrophages are central orchestrators of hepatic fibrogenesis [38] as proposed by the inhibition of the activation of HSCs in rat with suppressed macrophages infiltration [39] and the consequent reduction in fibrosis and inflammation in animal model of liver fibrosis depleted in macrophages [40, 41]. Upon liver injury, monocytes/macrophages are recruited. The recruitment process is mediated by chemokines and its receptors, especially C-C motif chemokine receptor 8 (CCR8) and 2 (CCR2) and CC chemokine ligand 2 (CCL2, also named monocyte chemotactic protein-1 (MCP-1)) [41–43]. Macrophages regulate inflammation and fibrosis by producing factors such as TGF-*β*, IL-1*β*, IL-8, PDGF, TNF-*α*, and MCP-1 [38]. These factors have proven to promote activation, proliferation, chemotaxis, extracellular matrix accumulation, and survival of myofibroblasts [44, 45]. Recently, macrophages have been described as mediators of the induction of liver fibrosis by activation of IkappaB kinase (IKK)/NF-*κ*B [46]. Inversely to their fibrogenic role in ongoing liver injury, macrophages have also a pivotal importance in liver repair [38, 47] since macrophages depletion during recovery leads to failure or retard of matrix degradation [40, 41]. Beneficial effects of macrophages during regeneration could be mediated by MMPs as well as others factors. Animals depleted with macrophages have sustained TIMPs messenger RNA expressions levels and reduced expression of MMP-2 and -13 [41]. Additionally, in a study where bone marrow-derived macrophages were delivered to animal model of advanced liver fibrosis, the macrophage therapy resulted in recruitment of host effectors cells, like endogenous macrophages and neutrophils, release of MMP-13 and -9, upregulation of anti-inflammatory IL-10, and reduction in hepatic myofibroblasts [48]. Macrophages can also favor liver fibrosis resolution by promoting HSCs apoptosis through TRAIL and MMP-9 production [49, 50]. These opposite effects of macrophages suggest that two distinct macrophage phenotypes mediate fibrogenesis and resolution [38, 47]. GR-1+ subset of hepatic macrophages could be associated for the profibrogenic effects [51].

### 2.6. Lymphocytes

Following liver injury, inflammatory lymphocytes infiltrate the hepatic parenchyma [52] as evidenced by the increase in the number of all liver lymphocytes subsequent to induction of fibrosis by CCl_4_ in mice. Lymphocytes take part in the development of liver fibrosis, especially CD8+ T cells [53]. Transgenic anti-inflammatory IL-10 from hepatocytes attenuates fibrosis through reduction of CD8+ T cells [54]. Once lymphocytes infiltrated the liver, they attach directly to activated HSCs to modulate fibrogenic response and induce lymphocytes proliferation. This interaction occurs because HSCs act as antigen-presenting cells by upregulating membrane proteins human leukocyte antigen II (HLA-II) and CD40 during fibrogenesis as well as major histocompatibility complex (MHC) class II and CD11c [53, 55]. A further* in vitro* study of intercellular interaction showed that CD8+ and CD4+ T-lymphocytes from peripheral blood lymphocytes of HBV/HCV-infected patients with advanced fibrosis can be engulfed by HSCs, a process that involves ICAM-1 and integrin molecules as well as Rac1 and Cdc42 pathways, resulting in HSCs activation and consequent fibrogenesis [56]. Additionally, CD4+ T cells can induce fibrogenesis by secreting cytokines, including TNF-*α* and IL-2 [57]. Interestingly, T helper subsets seem to play divergent role in fibrogenesis since C57BL/6 mice, displaying a Th1 lymphocytes response, have minimal fibrosis compared with BALB/c mice which exhibit Th2 response [58]. Th1 subset produces high level of antifibrotic IFN-*γ* whereas Th2 T cells produce high levels of profibrogenic cytokines mainly IL-4, IL-5, and IL-13 [59]. B lymphocytes are also involved in fibrogenesis; nevertheless its influence has been less investigated than T cells. Evidence of the profibrotic effect of B cells is that B cell-deficient mice have markedly reduced fibrosis than wild-type mice following CCl_4_ administration [60]. B cells can also induce fibrosis by producing profibrotic cytokine IL-6 [61].

### 2.7. Hepatic Stellate Cells (HSCs)

Almost all of the stimuli of inflammation converge to HSCs, which are not yet seen as a passive cell type in this process. Besides HSCs activation which is mediated by inflammatory species such as TGF-*β* and TNF-*α*, HSCs are involved in the recruitment of inflammatory cells through different mechanisms: by releasing chemokines and expressing cell adhesion molecules [62]. Chemokines are considered as the inflammatory mediators that modulate liver fibrosis by amplifying infiltration of inflammatory cells [63]. HSCs produce a wide range of profibrogenic and antifibrogenic chemokines and its receptors, including CCR2, CCR5, CCR7, CXCR3, CXCR4, CCL2 (MCP-1), CCL5 (also named regulated on activation, normal T cell expressed and secreted (RANTES)), CCL21, CXCL9, and CXCL10 (also named interferon gamma-inducible protein-10 (IP-10)) in schistosomiasis [64]. For example, CX3CL1/fractalkine soluble peptides produced by activated HSCs promote chemoattraction of monocytes and thus chronic inflammation of the liver, through CX3CR1 dependent signaling pathway [65]. Additionally, recruitment and migration of mononuclear cells within the perisinusoidal space of diseased livers might be the consequence of the interaction of HSCs with ICAM-1 and vascular cell adhesion protein-1 (V-CAM-1) ligand-bearing cells, such as lymphocyte function-associated antigen-1- or -Mac-1/very late activation antigen-4-positive inflammatory cells [66].

## 3. Inflammation and Immune Response as Targets of Antifibrotic Medicinal Plants

Since inflammation is a key process that contributes to the pathogenesis of liver fibrosis, to reduce inflammation and immune response is a relevant way to treat hepatic fibrosis [67, 68]. All the reviewed plants produce compounds that suppress inflammation, thereby highlighting anti-inflammatory properties as an important antifibrotic mechanism of medicinal plants. Even though all the reviewed medicinal plants have been unequally investigated, they share common anti-inflammatory mechanisms.

Acute or chronic administration of almost all the hepatotoxic/fibrogenic agents triggers an inflammatory response in liver; thus bioactive compounds or extracts from medicinal plants have been tested in different model of hepatotoxicity and fibrosis, including CCl_4_, TAA, MCD, high fat diet (HFD), ethanol, concanavalin A (ConA), dimethylnitrosamine (DMN), bile duct ligation (BDL), lipopolysaccharide (LPS), ischemia/reperfusion (I/R), and D-galactosamine (D-GalN). To facilitate the comprehension, we divided anti-inflammatory mechanisms of medicinal plants observed* in vivo* in hepatotoxic models which use a single administration of the hepatotoxic agent to induce inflammation, generally LPS, D-GalN, ConA, CCl_4_, and I/R (Table 1), and those which used a subacute or chronic administration of the hepatotoxic agent such as fibrotic models, including CCl_4_, DMN, TAA, MCD, HFD, and BDL (Table 2).

### 3.1. Cytokines

All the reviewed medicinal plants reduce liver fibrosis by downregulating hepatic expression and secretion into bloodstream of inflammatory cytokines, which is illustrated by reduced protein and mRNA expression levels into liver and decreased serum levels of cytokines, respectively. Inflammatory cytokines targeted by vegetal compounds include TNF-*α*, IL-1*α*, IL-1*β*, IL-2, IL-4, IL-6, IL-12, IL-18, and IFN-*γ*. Suppression of liver inflammation also involves the upregulation of anti-inflammatory cytokine IL-10 hepatic level and inhibition of inducible nitric oxide synthase (iNOS) and cyclooxygenase-2 (COX-2) expression.

### 3.2. Chemokines

Besides cytokines, bioactive compounds from reviewed medicinal plants regulate chemokines expression to suppress liver inflammation. Recruitment of immune cells to the injury site is orchestrated by chemokines such as CXCL10, MCP-1, MIP-1, and high mobility group protein B1 (HMGB1) and by other molecules like ICAM-1. During liver injury, expression of chemokines and their receptors is upregulated [69]. HMGB1 is released actively by monocytes/macrophages or passively by necrotic cells [70]. In hepatocytes, HMGB1 release is mediated by nuclear translocation of interferon regulatory factor 1 (IRF-1) [71]. Following injury, HMGB1 translocates to the cytoplasm and into the extracellular space where it acts as both a cytokine and a chemokine promoting inflammation through Toll-like receptor (TLR)2/TLR4 pathways [72]. Hepatic expression, secretion, and/or cytoplasmic translocation of HMGB1, especially from hepatocytes, are inhibited by curcumin from* C. longa*, glycyrrhizin from* G. glabra*, and chlorogenic acid from coffee [73–77]. Moreover, chlorogenic acid suppresses IRF-1 nuclear translocation [76]. MCP-1 targets monocytes and T-lymphocytes [78]. MCP-1 expression is decreased by curcumin, caffeine, and magnesium lithospermate B from* S. miltiorrhiza* [79–84]. ICAM-1 and CXCL10 mediate adhesion and migration of T-lymphocytes in the liver [85]. ICAM-1 expression is suppressed by curcumin, silymarin, and ginsenoside Rg1 from* P. ginseng* [80, 86–88], while expression of CXCL10 is inhibited by curcumin, ginsenoside Rg1, and sodium tanshinone IIA sulfonate from* S. miltiorrhiza* [86, 87, 89]. Moreover, leukocytes attractant chemokines MIP-2, MIP-1*α*, and RANTES are inhibited by curcumin and sodium tanshinone IIA sulfonate [79, 89]. Interestingly, MCP-1 and MIP-2, as well as TNF- *α*, IL-12, COX-2, and iNOS, are also inhibited* in vitro* by curcumin in isolated rat Kupffer cells treated with LPS [79] and HSCs stimulated with PDGF [90] suggesting that curcumin could selectively target these cells to reduce inflammation. As a result of chemokines downregulation, medicinal plants block the recruitment of immune cells into the liver. Curcumin, silymarin, extract of* G. biloba* (EGB), salvianolic acid B from* S. miltiorrhiza*, glycyrrhizin, picroliv from* P. kurroa*, ginsenoside Rg1, andrographolide from* A. paniculata*, and caffeine block neutrophils, lymphocytes, Kupffer cells, and mast cells hepatic infiltration following liver injury [81, 82, 86, 87, 91–102]. However, tanshinone from* S. miltiorrhiza* increases T lymphocyte subset CD3+, CD4+, and CD8+ ratios in ConA treated mice [103].

### 3.3. Nuclear Factor-Kappa B (NF-*κ*B)

NF-*κ*B is a transcription factor implicated in the regulation of a wide range of genes related to apoptosis, inflammation, and immune response. Expression of many chemokines, cytokines, and other inflammatory mediators, including iNOS, COX-2, MIP-2, MCP-1, IL-12, and TNF-*α*, is under control of NF-*κ*B activation. Following liver injury, NF-*κ*B is activated. This occurs via IKK-mediated phosphorylation and consequent degradation of inhibitory molecules, such as IkappaBalpha (I*κ*B*α*) and phosphorylation of p65 subunit of NF-*κ*B. The activated NF-*κ*B is then translocated into the nucleus where it binds to specific sequences of DNA to regulate gene expression of many inflammatory-related genes [104]. NF-*κ*B activity is inhibited by curcumin, silymarin, EGB, salvianolic acid B, polysaccharides, and sodium tanshinone IIA sulfonate from* S. miltiorrhiza*, glycyrrhizin, baicalin from* S. baicalensis*, saikosaponins A and D from* B. falcatum*,* P. urinaria koreanis*, 20(S)-ginsenoside Rg3, ginsenoside Rg1, and chlorogenic acid in different animal models [76, 79, 80, 82, 87–89, 105–118]. This occurs via suppressing NF-*κ*B nuclear translocation, downregulating phosphorylation, or increasing cytosolic level expression of inhibitory protein I*κ*B*α* or inhibiting nuclear expression or phosphorylation of NF-*κ*B p65 subunit. Moreover, polysaccharides from* S. miltiorrhiza* inhibit NF-*κ*B through upregulation of peroxiredoxin 6 (PRDX6) expression [105]. Inhibition of NF-*κ*B explains how medicinal plants reduce downstream induction of cytokines expression during liver fibrosis and suggests a common mechanism between their bioactive compounds. NF-*κ*B is regulated by the intracellular redox state; this implies that antioxidant compounds of reviewed medicinal plants reduce chronic liver injury-induced oxidative stress which is sensed by NF-*κ*B resulting in suppression of inflammation during liver fibrosis [119].

### 3.4. Toll-Like Receptors (TLRs)

Toll-like receptors, especially TLR2 and TLR4, are central mediators of the inflammation during liver fibrosis. TLRs ligands include pathogen-associated molecular patterns (PAMPs) as well as danger-associated molecular patterns (DAMPs) [120, 121]. DAMPs, such as HMGB1, are released as part of the fibrogenic cascade [122]. NF-*κ*B has been related to TLRs since stimulation of TLR2 leads to activation of NF-*κ*B through upregulation of myeloid differentiation primary response 88 (MyD88) [123]. In consequence, HMGB1-TLR2/TLR4-NF-*κ*B signaling pathway appears as a potential therapeutic target to suppress inflammation in liver fibrosis. In CCl_4_-induced liver fibrosis animal model and ConA challenged mice, curcumin inhibits liver expressions of TLR2, TLR4, and TLR9 [77, 91]. Baicalin and chlorogenic acid suppress TLR4-mediated inflammatory signaling pathway by reducing hepatic level of TLR4 and MyD88 protein expression in I/R-treated animal model of alcoholic fatty liver disease [116] and CCl_4_-fibrotic rats [76, 118], respectively. In these studies, disruption of TLR4 pathway correlated with downregulation of iNOS, COX-2, TNF-*α*, and IL-6 hepatic expression as well as NF-*κ*B inhibition [76, 116, 118]. Interestingly, chlorogenic acid also induces the liver expression of bone morphogenetic protein and activin membrane-bound inhibitor, the TGF-*β*1 pseudoreceptor, by downregulating TLR4, providing a link between proinflammatory and profibrogenic signals [118].

### 3.5. Heme Oxygenase-1 (HO-1)

Heme oxygenase-1 (HO-1) is a cytoprotective enzyme that is induced by a variety of stimuli, including cytokines, heavy metals, and oxidants [107]. HO-1 is transcriptionally regulated by the binding of redox-sensitive transcription factors, such as activator protein-1 (AP-1) and nuclear factor erythroid 2-related factor 2 (Nrf2), to antioxidant redox elements located in the promoter of the* ho-1* gene [124]. HO-1 exerts antioxidant, antiapoptotic, and anti-inflammatory functions following hepatic injuries [125]. The latter is mediated by inhibition of inflammatory response by targeting TNF-*α* and iNOS expression. Moreover, HO-1 induction reduces TLR4 overexpression and HMGB1 release [126, 127]. Hence, HO-1 could play a significant role in mediating anti-inflammatory properties of medicinal plants in liver fibrosis. Salvianolic acids A and B, glycyrrhizin, baicalin, 20(S)-ginsenoside Rg3, andrographolide, and chlorogenic acid increase HO-1 expression, level, and/or activity [76, 94, 106, 107, 109, 128–130]. Additionally, chlorogenic acid and silymarin induce the nuclear translocation of Nrf2 facilitating its binding with* ho-1* promoter [76, 131].

## 4. Conclusion

Medicinal plants could be a source of polyvalent antiliver fibrosis compounds targeting inflammation and the immune response. The importance of knowing the main mechanisms, by which medicinal plants act as antifibrotic agents, provides options for the development of pharmaceutical compounds and their subsequent use in medical practices. Since clinical studies are sparse and mainly use chronic hepatitis B and hepatitis C patients to assess the hepatoprotective effects of medicinal plants, more clinical proofs of their anti-inflammatory properties on patients with fibrosis induced by other agents than HBV and HCV are urgently needed.

## Figures and Tables

**Table 1:** Anti-inflammatory properties of medicinal plants observed in animals treated with acute administration of a hepatotoxic agent.

Plants	Bioactive compounds and/or extracts	Animals used	Hepatotoxic agents	Anti-inflammatory mechanisms
*C. longa *	Curcumin	Male BALB/c mice [86]	ConA	↓TNF-*α*, ↓IFN-*γ*, ↓IL-4, ↓ICAM-1, ↓CXCL10, and ↓CD4^+^
		Male BALB/c mice [91]	ConA	↓TNF-*α*, ↓IFN-*γ*, ↑IL-10, ↑TLR2, ↑TLR4, ↑TLR9, and ↓Kupffer cells
		Male C57BL/6 mice [73]	ConA	↓TNF-*α*, ↓IFN-*γ*, and ↓HMGB1
		Male BALB/c mice [74]	ConA	↓HMGB1, ↓TNF-*α*, ↓IL-1*β*, and ↓IL-6

*S. marianum *	Silymarin	Male C57BL/6 mice [132]	Ethanol	↓TNF-*α*

*G. biloba *	EGB	Male Wistar rats [133]	CCl_4_	↓TNF-*α* and ↓IL-6

*S. miltiorrhiza *	Polysaccharides	Kunming mice [105]	LPS + BCG	↓NF-*κ*B, ↑PRDX6, and ↓iNOS
	Sodium tanshinone IIA sulfonate	Male C57BL/6 mice [89]	ConA	↓TNF-*α*, ↓IFN-*γ*, ↓CXCL10, ↓MIP-1*α*, ↓RANTES, ↓STAT1, ↓NF-*κ*B, and ↑I*κ*B*α*
	Tanshinone IIA	Male Kunming mice [103]	ConA	↑CD3^+^, ↑CD4^+^, ↑CD8^+^, ↓IL-2, ↓IL-4, ↓IFN-*γ*, ↓TNF-*α*, and ↑IL-10
	Salvianolic acid A	Male SD rats [134]	CCl_4_	↓TNF-*α*

*G. glabra *	Glycyrrhizin	Male C57BL/6 (H-2b) mice [135]	ConA	↑IL-10
		Male ICR mice [128]	CCl_4_	↓TNF-*α*, ↓iNOS, ↓COX-2, and ↑HO-1
		Male BALB/c mice [136]	LPS + D-GalN	↓IL-18
		Male BALB/c mice [92]	ConA	↓iNOS, ↓neutrophils, and ↓macrophages
		Male SD rats [75]	I/R	↓HMGB1

*S. baicalensis *	Baicalin	Male BALB/c mice [137]	ConA	↓TNF-*α*, ↓IL-6, and ↓IFN-*γ*
		Male ICR mice [129]	CCl_4_	↓TNF-*α*, ↓iNOS, and ↑HO-1
		BALB/c mice [106]	LPS + D-GalN and TNF-*α* + D-GalN	↓TNF-*α*, ↓NF-*κ*B p65, and ↑HO-1
		Male SD rats [107]	I/R	↓TNF-*α*, ↓IL-6, ↓iNOS, ↓COX-2, ↑HO-1, ↓NF-*κ*B p65, and ↑I*κ*B*α*

*Phyllanthus *species	Ethanolic extract of fruits from *P. emblica *	Male Wistar rats [138]	Ethanol	↓IL-1*β*
	*P. urinaria koreanis *	C57BL/6 mice [108]	MCD	↓TNF-*α*, ↓IL-6, ↓p-JNK, and ↓NF-*κ*B p65
	Ethanolic extract of *P. rheedii *	Wistar rats [139]	D-GalN	↓TNF-*α*
	Aqueous extract of *P. amarus *	Male Wistar rats [140]	Ethanol	↓TNF-*α*

*B. aristata *	Berberine	Male BALB/c mice [141]	CCl_4_	↓TNF-*α*, ↓iNOS, and ↓COX-2

*P. kurroa *	Picroliv	Male SD rats [93]	I/R	↓IL-1*α*, ↓IL-1*β*, and ↓neutrophils

*Ginseng *species	20(S)-ginsenoside Rg3	Male Wistar rats [109]	LPS	↓NF-*κ*B p65, ↓iNOS, ↓COX-2, and ↑HO-1
	Ginsenoside Rd	Male ICR mice [142]	LPS	↓iNOS and ↓COX-2
	Ginsenoside Rg1	Male C57BL/6 mice [87]	ConA	↓TNF-*α*, ↓IFN-*γ*, ↓IL-6, ↓NF-*κ*B, ↑I*κ*B*α*, ↓NF-*κ*B p65, ↓CD4^+^, ↓CD8^+^, ↓ICAM-1, and ↓CXCL10

*A. paniculata *	andrographolide	Male C57BL/6 mice [94]	CCl_4_	↓TNF-*α*, ↑HO-1, and ↓lymphocytes

Coffeespecies	Caffeine, nicotinic acid, and nonsubstituted pyrazinoic acid	Male Wistar rats [143]	LPS + D-GalN	↓TNF-*α* and ↑IL-10
	Chlorogenic acid	Male SD rats [76]	I/R	↓TNF-*α*, ↓iNOS, ↓COX-2, ↑HO-1, ↑Nrf2, ↓NF-*κ*B p65, ↓HMGB1, ↓IRF-1, and ↓TLR4

Note: ↓: inhibitory effect, ↑: inductor effect, BCG: Bacille Calmette-Guérin, CCl_4_: carbon tetrachloride, CD3^+^: cluster of differentiation 3 T-lymphocytes, CD4^+^: cluster of differentiation 4 T-lymphocytes, CD8^+^: cluster of differentiation 8 T-lymphocytes, ConA: concanavalin, COX-2: cyclooxygenase-2, CXCL10: interferon gamma-induced protein-10, D-GalN: D-galactosamine, EGB: *G. biloba *extract, standardized extract of the leaves of *G. biloba*, a mixture mainly composed of flavonoid glycosides (24%) and terpenoides (6%), including ginkgolides and bilobalide, HMGB1: high mobility group protein B1, HO-1: heme oxygenase-1, I/R: ischemia/reperfusion, I*κ*B*α*: IkappaBalpha (NF-*κ*B inhibitor), ICAM-1: intracellular adhesion molecule-1, IFN-*γ*: interferon gamma, IL: interleukin, iNOS: inducible nitric oxide synthase, IRF-1: interferon regulatory factor 1, LPS: lipopolysaccharides, MCD: methionine and choline deficient diet, MIP-1*α*: macrophage inflammatory protein-1 alpha, NF-*κ*B: nuclear factor kappaB, NF-*κ*B p65: p65 subunit of nuclear factor kappaB, Nrf2: nuclear factor erythroid 2-related factor 2, p-JNK: phosphorylated c-Jun N-terminal kinases, PRDX6: peroxiredoxin 6, RANTES: regulated on activation, normal T cell expressed and secreted, SD: Sprague-Dawley, STAT1: signal transducers and activators of transcription 1, TLR: Toll-like receptor, and TNF-*α*: tumor necrosis factor alpha.

**Table 2:** Anti-inflammatory properties of medicinal plants observed in animals treated with a subacute or chronic administration of a hepatotoxic agent.

Plants	Bioactive compounds and/or extracts	Animals used	Profibrogenic agents	Anti-inflammatory mechanisms
*C. longa *	Curcumin	Male Wistar rats [79]	Fish oil + ethanol	↓NF-*κ*B, ↑I*κ*B*α*, ↓TNF-*α*, ↓IL-12, ↓MCP-1, ↓MIP-2, ↓COX-2, and ↓iNOS
		Female C57BL6/J mice [80]	MCD	↓NF-*κ*B, ↓ICAM-1, ↓COX-2, and ↓MCP-1
		Male C57BL6/J mice [81]	MCD	↓MCP-1 and ↓CD11b
		Male SD rats [144]	CCl_4_	↓IFN-*γ*, ↓TNF-*α*, and ↓IL-6
		Male SD rats [77]	CCl_4_	↓TNF-*α*, ↓IL-6, ↓MCP-1, ↓HMGB1, ↓TLR4, and ↓TLR2
		SD rats [110]	CCl_4_	↓NF-*κ*B, ↓TNF-*α*, ↓IL-1*β*, ↓IL-6, and ↑IL-10
		Male C57BL6/J mice [145]	CCl_4_	↓IL-6 and ↓TNF-*α*
		Rats [111]	CCl_4_	↓NF-*κ*B, ↓IL-6, and ↓TNF-*α*
		Male BALB/c mice [95]	TAA	↓TNF-*α* and ↓macrophages
		Male *ob/ob* C57BL/6J mice [82]		↓TNF-*α*, ↓IL-6, ↓MCP-1, ↓NF-*κ*B, and ↓macrophages

*S. marianum *	Silymarin	Male Wistar rats [96]	CCl_4_	↓mast cells
		Male SD rats [88]	DMN	↓iNOS, ↓ICAM-1, and ↓NF-*κ*B
		Male OLETF rats [131]	MCD	↓TNF-*α*, ↑Nrf2, and ↓ERK
		Male CD-1 Swiss albino mice [97]	*S. mansoni *	↓mast cells

*G. biloba *	EGB	Rats [112]	HFD	↓NF-*κ*B p65, ↓NF-*κ*B
		Male Wistar albino rats [98]	BDL	↓TNF-*α* and ↓neutrophils
		Male SD rats [146]	Ethanol	↓TNF-*α*
		Rats [113]	HFD	↓NF-*κ*B p65

*S. miltiorrhiza *	*S. miltiorrhiza *	Male SD rats [147]	CCl_4_	↓NOSII
		Male Kunming mice [148]	Iron dextran	↓TNF-*α* and ↓IL-1*α*
	Salvianolic acid A and salvianolic acid B	Male SD rats [130]	TAA	↓HO-1, ↓iNOS, ↓TNF-*α*, ↓IL-6, and ↓IL-1*β*
	Salvianolic acid B	SD rats [99]	CCl_4_	↓TNF-*α* and ↓CD14
		Male SD rats [114]	CCl_4_	↓NF-*κ*B, ↑I*κ*B*α*

*G. glabra *	Glycyrrhizin	Male SD rats [115]	CCl_4 _+ ethanol	↓NF-*κ*B
		Female BALB/c mice [100]	ConA	↓Th1, ↓Th2, ↓Th17, ↓Treg, ↑IFN-*γ*, and ↑IL-10

*S. baicalensis *	Baicalin	Male SD rats [149]	CCl_4_	↓TNF-*α*, ↓IL-6, and ↑IL-10
		Male SD rats [116]	Ethanol + I/R	↓TLR-4, ↓MyD88, ↓NF-*κ*B p65, ↑I*κ*B*α*, ↓TNF-*α*, ↓IL-6, ↓iNOS, and ↓COX-2

*B. falcatum *	Saikosaponin D	Male SD rats [117]	CCl_4_	↓TNF-*α*, ↓IL-6, ↓NF-*κ*B p65, and ↑I*κ*B*α*
	Saikosaponin A	Male SD rats [110]	CCl_4_	↓NF-*κ*B, ↓TNF-*α*, ↓IL-6, ↓IL-1*β*, and ↑IL-10

*B. aristata *	Berberine	Mice [150]	CCl_4_	↓TNF-*α*

*Ginseng *species	*P. notoginseng *saponins	Male SD rats [151]	CCl_4_	↓TNF-*α*, ↓IL-6, and ↑IL-10

*A. paniculata *	Andrographolide	Male SD rats [152]	BDL	↓TNF-*α* and ↓IL-1*β*
		Male SD rats [101]	Copper sulfate	↓TNF-*α* and ↓Kupffer cells

Coffeespecies	Coffee	Male SD rats [153]	DMN	↓TNF-*α*, ↓IL-1*β*, and ↓iNOS
	Caffeine	Male Kunming mice [83]	Ethanol	↓TNF-*α*, ↓IL-6, ↓MCP-1, ↓IFN-*γ*, and ↓IL-1*β*
		Male SD rats [102]	TAA	↓inflammatory cells
	Decaffeinated coffee, polyphenols, and melanoidins	Male Wistar rats [154]	HFD	↓TNF-*α*, ↑IL-6, ↓IFN-*γ*, ↓IL-1*α*, ↓IL-1*β*, ↑IL-4, ↑IL10, and ↑adipo-R2
	Chlorogenic acid	SD rats [118]	CCl_4_	↓TLR4, ↓MyD88, ↓iNOS, ↓COX-2, ↑Bambi, ↓TNF-*α*, ↓IL-6, ↓IL-1*β*, ↑I*κ*B*α*, and ↓NF-*κ*B

Note: ↓: inhibitory effect, ↑: inductor effect, adipo-R2: adiponectin receptor 2, Bambi: bone morphogenetic protein and activin membrane-bound inhibitor, BDL: bile duct ligation, CCl_4_: carbon tetrachloride, CD11b: cluster of differentiation 11b, CD14: cluster of differentiation 14, ConA: concanavalin, COX-2: cyclooxygenase-2, DMN: dimethylnitrosamine, EGB: *G. biloba *extract, standardized extract of the leaves of *G. biloba*, a mixture mainly composed of flavonoid glycosides (24%) and terpenoides (6%), including ginkgolides and bilobalide, ERK: extracellular signal regulated kinases, HFD: high fat diet, HMGB1: high mobility group protein B1, HO-1: heme oxygenase-1, I/R: ischemia/reperfusion, I*κ*B*α*: IkappaBalpha (NF-*κ*B inhibitor), ICAM-1: intracellular adhesion molecule-1, IFN-*γ*: interferon gamma, IL: interleukin, iNOS: inducible nitric oxide synthase, MCD: methionine and choline deficient diet, MCP-1: monocyte chemotactic protein-1, MIP-2: macrophage inflammatory protein-2, MyD88: myeloid differentiation primary response 88, NF-*κ*B: nuclear factor kappaB, NF-*κ*B p65: p65 subunit of nuclear factor kappaB, NOSII: nitric oxide synthase II, Nrf2: nuclear factor erythroid 2-related factor 2, OLETF: Otsuka Long Evans Tokushima Fatty, SD: Sprague-Dawley, TAA: thioacetamide, Th1: T helper 1, Th2: T helper 2, Th17: T helper 17, Treg: regulatory T cells, TLR: Toll-like receptor, and TNF-*α*: tumor necrosis factor alpha.
